# Supplementary material for: Machining water through laser cutting of nanoparticle-encased water pancakes
Source: Nat Commun. 2023 Jun 29;14:3853. doi: 10.1038/s41467-023-39574-3 (PMC10310854; doi:10.1038/s41467-023-39574-3)
Supplement: Supplementary file 1 — Supplementary information [file 41467_2023_39574_MOESM1_ESM.pdf]

Supplementary information

Machining water through laser cutting of nanoparticle-encased water pancakes

Jicheng Niu[1,2], Wenjing Liu[3], Jasmine Xinze Li[2], Xianglong Pang[3], Yulin Liu[1,2], Chao Zhang[1,2], Keyang Yue[1,2], Yulin Zhou[1,2], Feng Xu[1,2], Xiaoguang Li*[3] and Fei Li*[,1,2]

[1] *The Key Laboratory of Biomedical Information Engineering of Ministry of Education, School of Life Science and Technology, Xi'an Jiaotong University, Xi'an 710049, P.R. China*

[2] *Bioinspired Engineering and Biomechanics Center (BEBC), Xi'an Jiaotong University, Xi'an 710049, P.R. China*

[3] *Shaanxi Basic Discipline (Liquid Physics) Research Center, School of Physical Science and Technology, Northwestern Polytechnical University, Xi'an, China*

**Corresponding authors: lixiaoguang@nwpu.edu.cn; feili@mail.xjtu.edu.cn*

Table of Contents

Supplementary Figure 1.......... S4

Supplementary Figure 2.......... S4

Supplementary Figure 3.......... S5

Supplementary Figure 4.......... S5

Supplementary Figure 5.......... S6

Supplementary Figure 6.......... S6

Supplementary Figure 7.......... S7

Supplementary Figure 8.......... S7

Supplementary Figure 9.......... S8

Supplementary Figure 10.......... S8

Supplementary Figure 11.......... S9

Supplementary Figure 12.......... S9

Supplementary Figure 13.......... S10

Supplementary Figure 14.......... S10

Supplementary Figure 15.......... S11

Supplementary Note 1.......... S12

Supplementary Note 2.......... S12

Supplementary Note 3.......... S12

Supplementary Note 4.......... S12

Supplementary Note 5.......... S13

Supplementary Note 6.......... S13

Supplementary Note 7.......... S13

Supplementary Note 8.......... S13

Supplementary Note 9.......... S14

Supplementary Note 10.......... S14

Supplementary Note 11.......... S14

Supplementary Note 12.......... S15

Supplementary Note 13.......... S15

Supplementary Note 14.. S15

Supplementary Note 15.. S15

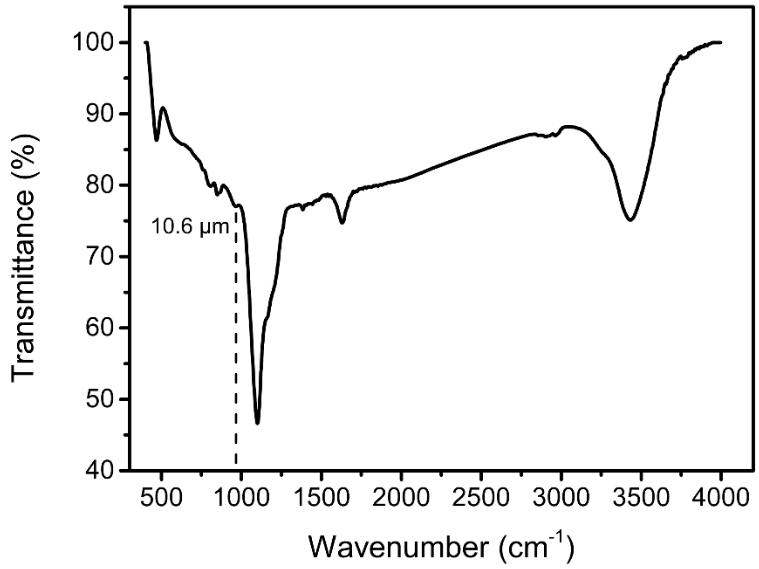

Supplementary Figure 1. Infrared spectra of the silica nanoparticles. The dashed line indicates the position of the wavelength of the laser used in laser cutting of nanoparticle-encased water pancakes.

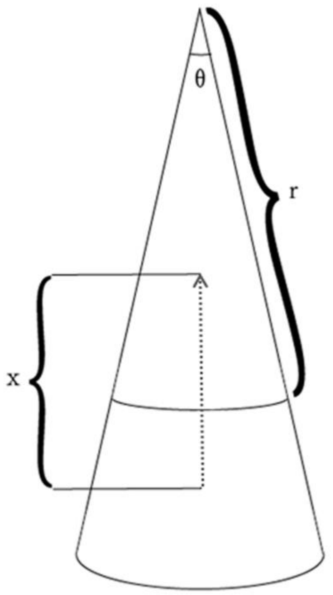

Supplementary Figure 2. Gravity center movement of water replenishment process. Schematic diagram of gravity center movement of water replenishment process in theoretical analysis.

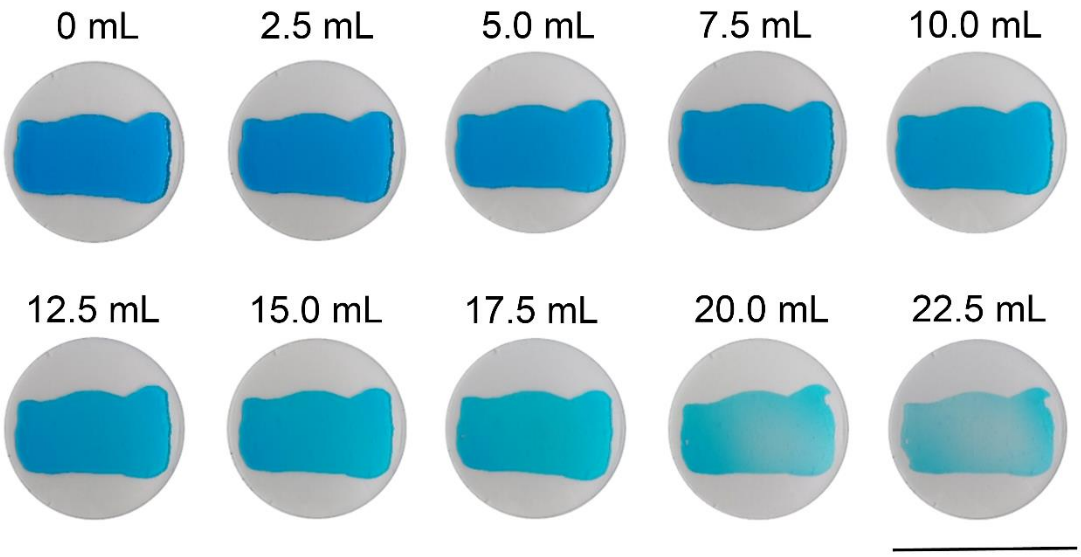

Supplementary Figure 3. Effect of the volume of pumped water on the area (top view) of nanoparticle-encased water pancake. First, 25.0 mL of brilliant blue pigment solution with a concentration of 0.5 mg mL^{-1} was injected into the nanoparticle-encased water pancake. Then, 2.5 mL of brilliant blue pigment solution were extracted from the nanoparticle-encased water pancake each time. The scale bar represents 15 cm.

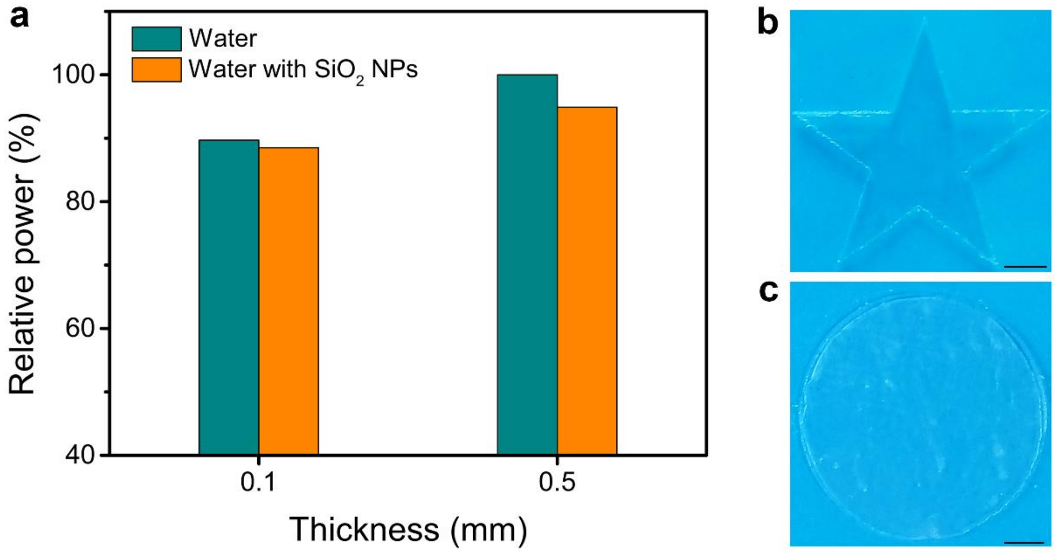

Supplementary Figure 4. Machining of light-absorbing liquid in nanoparticle-encased water pancake through laser cutting. (a) Comparison of laser power required for laser cutting between pure water and water containing silica nanoparticles. (b) Pentagram pattern processed by laser cutting. (c) Circular pattern processed by laser cutting. The scale bars represent 5 mm.

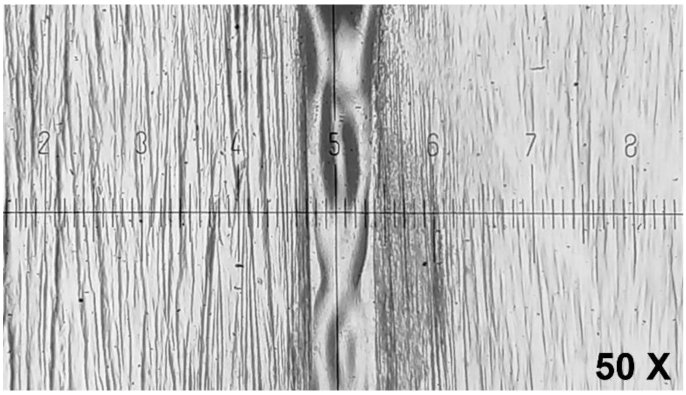

Supplementary Figure 5. Characterization of machining accuracy. Optical microscope photograph of a slit created by laser cutting of nanoparticle-encased water pancakes and a microscope eyepiece scale. The width of the slit enlarged by 50 folds is about 1 cm. The laser cutting system was equipped with a focusing lens with a focal length of 50.8 mm.

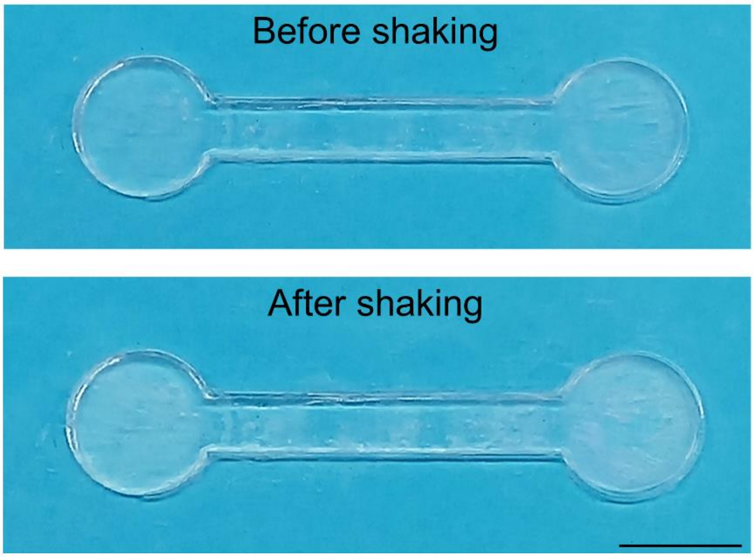

Supplementary Figure 6. Characterization of self-supporting chips stability. Photos of the self-supporting chips with and without shaking on a shaper with a shaking frequency of 100 rpm. The scale bar represents 1 cm.

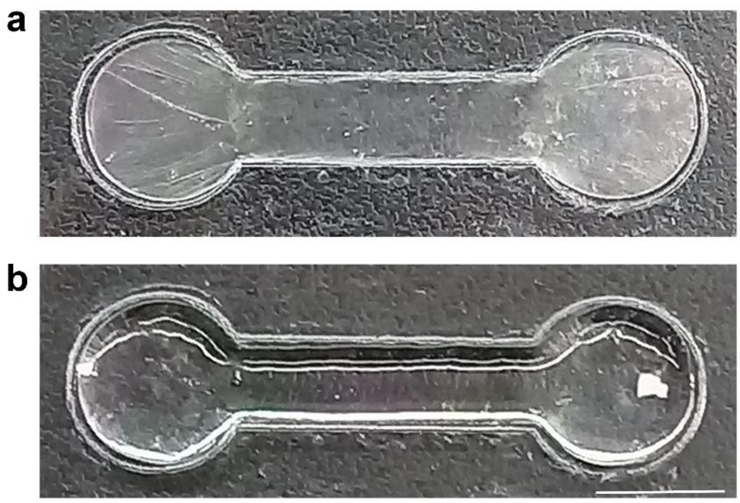

Supplementary Figure 7. Demonstration of openness of self-supporting chips. a, b, Photographs of a self-supporting chip (**a**) fabricated by laser cutting and (**b**) then filled with water. The scale bar represents 1 cm.

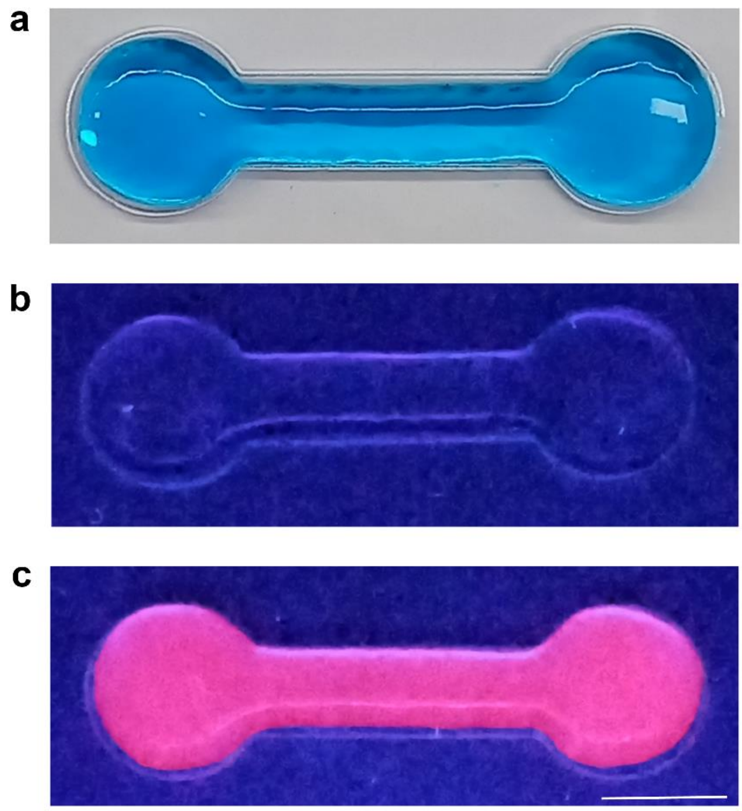

Supplementary Figure 8. Demonstration of transparency of self-supporting chips. **a-c**, Photographs of self-supporting chips filled with (**a**) 0.5 mg mL^{-1} brilliant blue pigment solution (**b**) water under 365 nm UV light and (**c**) 1.0 mg mL^{-1} phycocyanin solution under 365 nm UV light. The scale bar represents 1 cm.

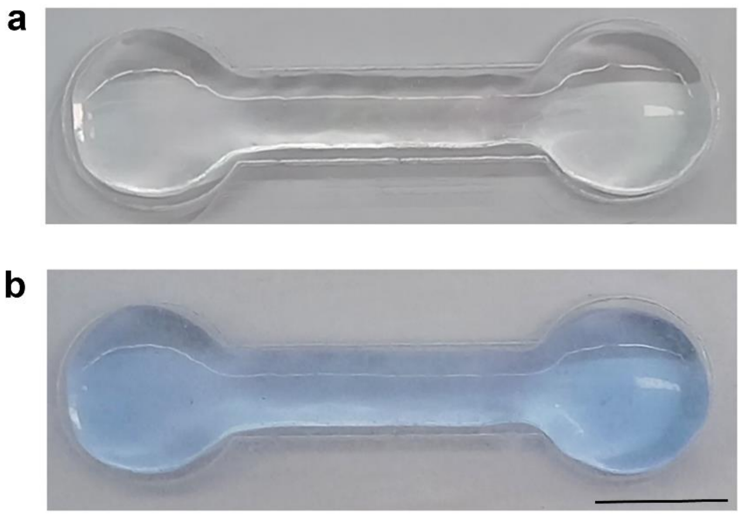

Supplementary Figure 9. Demonstration of breathability of self-supporting chips. **a, b**, Photographs of the self-supporting chips filled with 10 mM $CuSO_4$ solution (**a**) in air and (**b**) under ammonia gas. The scale bar represents 1 cm.

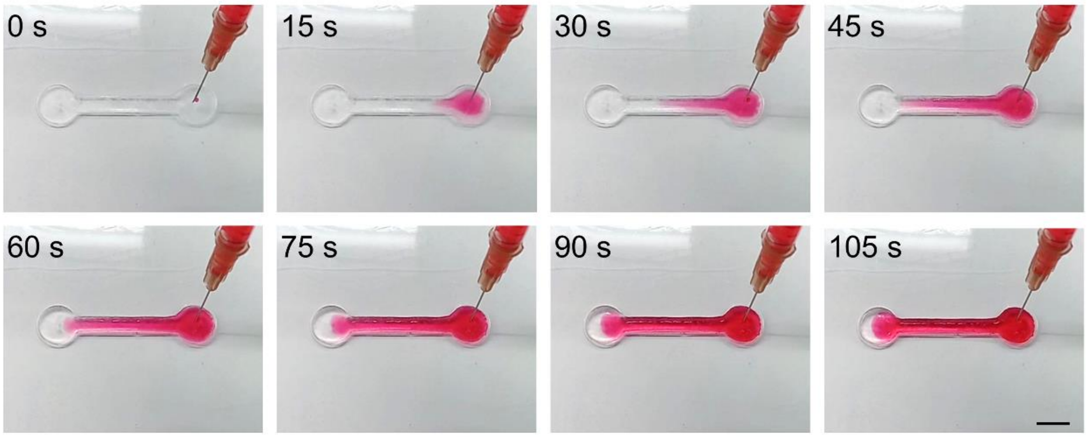

Supplementary Figure 10. Self-driving liquid flow within self-supporting chip. Photographs of the flow process of red liquid driven by the pressure provided by the own gravity of liquid in a self-supporting chip. The scale bar represents 1 cm.

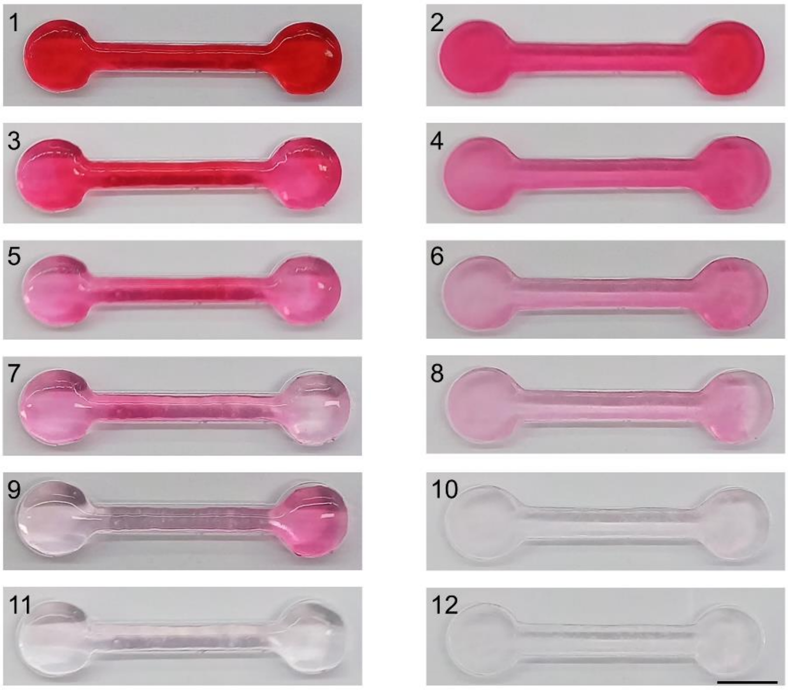

Supplementary Figure 11. Reuse of self-supporting chips. Photographs of the replacement process of red liquid in a self-supporting chip with water by rinses for multiple times. The scale bar represents 1 cm.

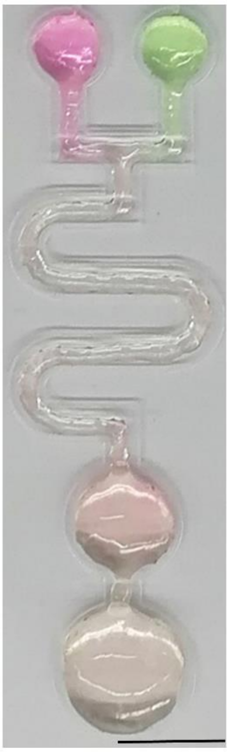

Supplementary Figure 12. Mixing of highly viscous samples in self-supporting chips. Photograph of two dyes (red and green) dissolved in 80% glycerol mixed in a self-supporting chip. The scale bar represents 1 cm.

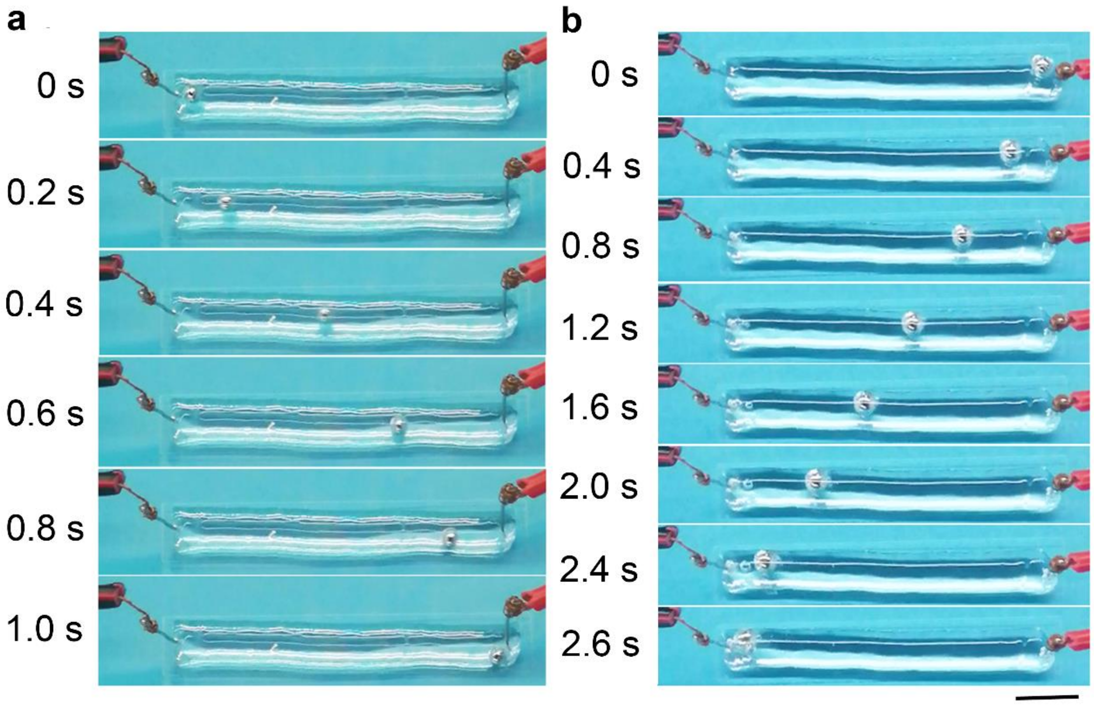

Supplementary Figure 13. Effect of pH of solution on electrokinetic manipulation of liquid metal. a, Dynamic illustration of electrokinetic migration of liquid metal in a self-supporting chip contained 50 mM NaOH (DC voltage: 50 V). **b**, Dynamic illustration of electrokinetic migration of liquid metal in a self-supporting chip contained 50 mM HCl solution (DC voltage: 50 V). The scale bar represents 1 cm.

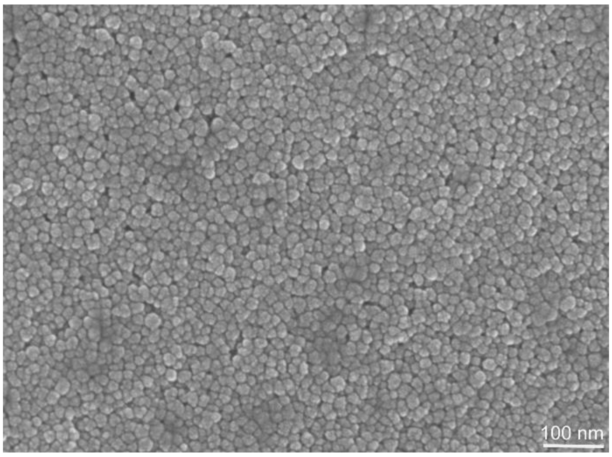

Supplementary Figure 14. Characterization of morphology of silica nanoparticles using scanning electron microscopy. Scanning electron microscopy image of silica nanoparticles.

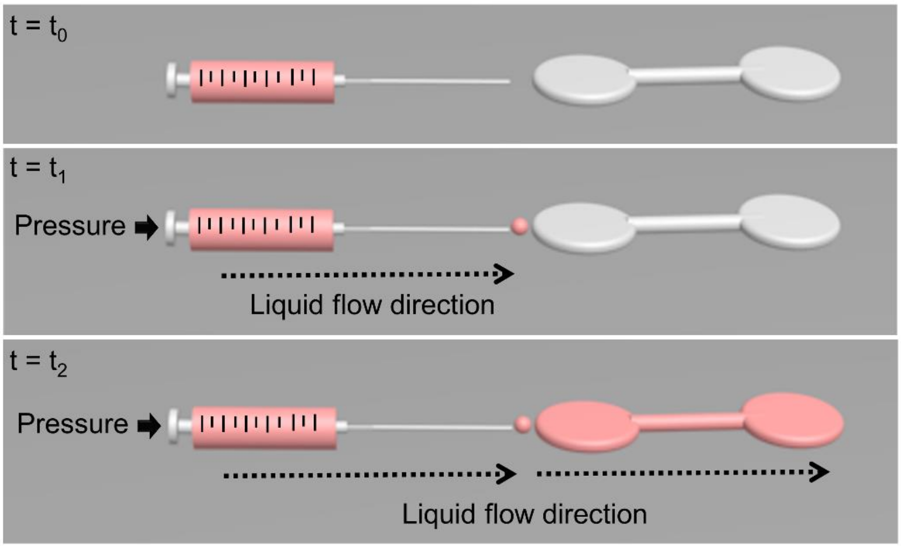

Supplementary Figure 15. Liquids pumping using the syringe pump. Schematic diagram of connection of the syringe pump to the self-supporting chip during pumping liquids.

Supplementary Note 1: Infrared spectra of the silica nanoparticles

To test the absorption ability of the silica nanoparticles used in our work for infrared light with a wavelength of 10.6 μm, we characterized the infrared spectra of the silica nanoparticles collected from the nanoparticle-encased water pancake in the wavelength range of 2.5–20.0 μm (Supplementary Figure 1).

Supplementary Note 2: Gravity center movement of water replenishment process in theoretical analysis

In theoretical analysis, the deformation and flow of water under pressure water replenishment process was simplified as the movement of the center of mass of rigid body under uniform pressure (Supplementary Figure 2). For a water replenishment process with incision radius r, the moving distance of the center of gravity x and r satisfy equation (S1).

$$x = \frac{\sqrt{6} - \sqrt{2}}{2} r \qquad \text{(S1)}$$

Supplementary Note 3: Variation of top view area of nanoparticle-encased water pancake (NEWP) with water being pumped out

To explore the dynamic changes in the area of nanoparticle-encased water pancake during extraction of water from nanoparticle-encased water pancake, a nanoparticle-encased water pancake containing 25 mL brilliant blue pigment solution was fabricated. Supplementary Figure 3 presents the photographs of the fabricated nanoparticle-encased water pancake by pumping 2.5 mL brilliant blue pigment solution each time.

Supplementary Note 4: Machining of light-absorbing liquid in nanoparticle-encased water pancake through laser cutting

To explore whether the same mechanism works when the liquid is light absorbing, the water used to make the nanoparticle-encased water pancake was added with 0.2 g L^{-1} of water-soluble SiO_2 nanoparticles which absorb infrared light with a wavelength of 10.6 μm. The comparison of laser power required for cutting between water and water containing silica nanoparticles is shown in Supplementary Figure 4a, from which we

can see that the laser cutting method proposed in this study also works for the light-absorbing liquids (Supplementary Figure 4b,c).

Supplementary Note 5: Photograph of a slit created by laser cutting of nanoparticle-encased water pancakes

To investigate whether a smaller spot can achieve a smaller slit, we adjusted the focal length of the laser cutting system through using a focusing lens with a smaller focal length (50.8 mm) to obtain a smaller laser spot. We can see that the slits with a width of 200 μm can be achieved through using the focusing lens with a smaller focal length (Supplementary Figure 5).

Supplementary Note 6: Stability of self-supporting chips during transfer

To explore the stability of the self-supporting chips during transfer, the self-supporting chips were placed on a shaker with a speed of 100 rpm. The photos of the self-supporting chips with and without shaking are shown in Supplementary Figure 6.

Supplementary Note 7: Openness of self-supporting chip

As shown in Supplementary Figure 7a, the surface of the initial laser-cutting fabricated self-supporting chip was covered with a thin film through jamming silica nanoparticles, which was visible to naked eyes. The jamming of silica nanoparticles was caused by reduction in the surface area of water during pumping, in which the dynamic process of nanoparticles jamming and dispersion varied with the surface area of the encapsulated water. Therefore, the initial self-supporting chip with nanoparticle shell had enough space for subsequent liquid injection. During the process of injecting water into the self-supporting chip, the jammed nanoparticles gradually dispersed as the surface area of the water increased, then the self-supporting chip become transparent (Supplementary Figure 7b).

Supplementary Note 8: Transparency of self-supporting chips

To develop the self-supporting chip as an analytical platform combining colorimetric and fluorescent detection, transparency of the self-supporting chips is critical. As shown

in Supplementary Figure 8a, thanks to the good transparency of self-supporting chips after filling with liquid, the colorimetric signals in self-supporting chips were easy to acquire. For fluorescence detection, not only light transmittance is of great importance, but also low fluorescence background is critical. As presented in Supplementary Figure 8b, the fluorescence background of the self-supporting chip was very weak under the irradiation of ultraviolet light with a wavelength of 365 nm when the self-supporting chip is filled with water. Under the irradiation of 365 nm UV light, the obvious red fluorescence can be detected after the self-supporting chip was injected with the phycocyanin solution (Supplementary Figure 8c).

Supplementary Note 9: Breathability of self-supporting chips

A cupro-ammonium complexation reaction was designed to study the gas permeability of self-supporting chips. As a control experiment, initial self-supporting chip was injected with copper sulfate solution with a concentration of 10 mM and exposed to air. After standing for 10 minutes, the self-supporting chip showed no obvious color change, as shown in Supplementary Figure 9a. In the petri dish, 15% ammonia water was then dripped around the self-supporting chip with 10 mM of copper sulfate solution. Supplementary Figure 9b provides a photo of the self-supporting chip after 2.5 minutes of reaction, in which a distinct blue color caused by the cupro-ammonium complexation reaction can be observed. The above results indicate that the ammonia gas successfully reacted with the copper sulfate in the self-supporting chip, confirming the gas permeability of the self-supporting chip.

Supplementary Note 10: Self-driving liquid flow within self-supporting chip

For the self-supporting chips, the flow of the liquid can be driven by the pressure provided by the liquid's gravity. The self-driven flow process of the red liquid dropped through a syringe, in which the red liquid was dropped vertically into the self-supporting chips as demonstrated in Supplementary Figure 10.

Supplementary Note 11: Reuse of self-supporting chips

The self-supporting chips can be reused after performing liquid manipulation

experiments. The replacement of the red liquid in the self-supporting chips with purified water by multiple rinses, in which rinsing of the self-supporting chip was performed by sequentially injecting pure water into the chip and sucking liquid from the chip (Supplementary Figure 11).

Supplementary Note 12: Mixing of highly viscous samples in self-supporting chips

Two dyes (red and green) dissolved in 80% glycerol were used to verify the ability of self-supporting chips to mix high viscosity samples. As presented in Supplementary Figure 12, the red and green dyes were successfully mixed based on the action of the mixed liquid in the tortuous channel in the self-supporting chip.

Supplementary Note 13: Electrokinetic manipulation of liquid metal in self-supporting chips

A pillar-shaped self-supporting chip was used to study electrokinetic manipulation of liquid metals. Supplementary Figure 13a shows the dynamic process of liquid metal migration driven by an electric field when the self-supporting chip was filled with sodium hydroxide solution, in which liquid metal beads migrated from cathode to anode within 1 s. However, the migration of liquid metal beads from anode to cathode was achieved within 2.6 s when the self-supporting chip was hydrochloric acid solution, as shown in Supplementary Figure 13b. The above results confirm that the liquid metal exhibits different charges when placed in an alkaline or acidic solution, with which the movement of the liquid metal can be manipulated using an electric field.

Supplementary Note 14: Characterization of morphology of silica nanoparticles using scanning electron microscopy.

To demonstrate the size of the silica nanoparticles used in this work, we characterized the morphology of silica nanoparticles collected from the nanoparticle-encased water pancake using scanning electron microscopy (Supplementary Figure 14).

Supplementary Note 15: Liquids pumping using the syringe pump

The tygon hose connected to the syringe pump was not inserted inside the self-supporting chips, while it was fixed near the injection port of self-supporting chips, during the pumping of liquid into the self-supporting chips using the syringe pump (Supplementary Figure 15). Benefiting from the openness of self-supporting chips, the syringe pump-driven fluid can still enter the self-supporting chips after being in contact with self-supporting chips.
