# Supplementary material for: Machining water through laser cutting of nanoparticle-encased water pancakes
Source: Nat Commun. 2023 Jun 29;14:3853. doi: 10.1038/s41467-023-39574-3 (PMC10310854; doi:10.1038/s41467-023-39574-3)
Supplement: Supplementary file 3 — Description of Additional Supplementary Information [file 41467_2023_39574_MOESM3_ESM.pdf]

Description of Additional Supplementary Files

Supplementary Movie 1: Simulation of water shape and temperature change in nanoparticle-encased water pancake (NEWP) during laser cutting. The initial thicknesses of the water in NEWP was 0.25 mm.

Supplementary Movie 2: Simulation of water shape and temperature change in nanoparticle-encased water pancake (NEWP) during laser cutting. The initial thicknesses of the water in NEWP was 1.00 mm.

Supplementary Movie 3: Dynamic process of the self-supporting chip fabricated through laser cutting of nanoparticleencased water pancakes (LCNEWP). The video runs in real time.

Supplementary Movie 4: Stability of self-supporting chip during shaking. The video runs in real time.

Supplementary Movie 5: Red liquid pumping within a self-supporting chip. The video runs in real time. Supplementary Movie.

Supplementary Movie 6: Flow of red liquid driven by its own gravity within a self-supporting chip. The video runs in real time.

Supplementary Movie 7: Red liquid pumping within a self-supporting chip, in which the fluid channel was isolated from the injection reservoir by cutting. The video runs in real time.

Supplementary Movie 8: Red liquid pumping within a self-supporting chip, in which the injection reservoir and fluidic channel were reconnected with droplets. The video runs in real time.

Supplementary Movie 9: Mixing of various liquids (blue, green and red liquids) in a selfsupporting chip with radial-array fluid channels. The video runs in real time.

Supplementary Movie 10: Mixing of two liquids (green and red liquids) in a self-supporting chip with single curved fluid channel. The video runs in real time.

Supplementary Movie 11.: Concentration gradient generated in a self-supporting chip with an array of curved fluid channels (green and red liquids). The video runs in real time.

.

Supplementary Movie 12.: Concentration gradient generated in a selfsupporting chip with a “Y” fluid channel (green and red liquids). The video runs in real time.

Supplementary Movie 13: Segmented solution (blue, green and red liquids) generated in a self-supporting chip with a spiral channel. The video runs in real time.

Supplementary Movie 14: Electrokinetic manipulation of liquid metal in labyrinth-shaped self-supporting chip filled with 100 mM NaOH solution. The liquid metal migrated from cathode to anode driven by an electric field force. The video runs in real time.

Supplementary Movie 15: Electrokinetic manipulation of liquid metal in a labyrinthshaped self-supporting chip filled with 100 mM NaOH solution. The liquid metal migrated from cathode to anode driven by an electric field force. The video runs in real time.

Supplementary Movie 16: Demonstration of lighting a LED in a current loop consisting of a patterned conductive hydrogel, liquid metal, LED and a DC power source. The red women and blue men were patterned conductive hydrogels. When the power supply was connected, the LED in the loop was successfully lit up. The video runs in real time.
